# Bovine leptospirosis: effects on reproduction and an approach to research in Colombia

**DOI:** 10.1007/s11250-022-03235-2

**Published:** 2022-08-09

**Authors:** Agustín Góngora Orjuela, Jorge L. Parra-Arango, Luz A. Sarmiento-Rubiano

**Affiliations:** 1 Grupo de Investigación en Reproducción y Genética Animal (Reproduction and Animal Genetics Research Group, GIRGA), Universidad de los Llanos, Villavicencio, Meta Colombia; 2 Universidad de los Llanos, Villavicencio, Meta Colombia; 3 Universidad Metropolitana, Barranquilla, Atlántico Colombia

**Keywords:** *Leptospira*, Abortion, Public health, Serovar, Zoonosis

## Abstract

Leptospirosis is the most widespread zoonosis worldwide, causing severe effects on beef and dairy cattle farming and other livestock. Colombia geographical location in the tropical zone, high biodiversity, and climatic conditions promote *Leptospira* growth and prevalence. This review article presents state-of-the-art knowledge about the effects of leptospirosis on bovine reproduction and a critical analysis of the research carried out in Colombia. The analysis of the information allows us to infer a sustained increase in prevalence over the last decade in the densest livestock production areas and a high serovar diversity of circulating pathogenic *Leptospira*. Given the zoonotic nature of leptospirosis, an inter-institutional effort is required to implement prevention, control, and monitoring programs under one-health concept.

## Introduction

Leptospirosis is an infectious disease that causes large economic losses in the cattle industry (Adler 2015). It is one of the most widespread zoonotic diseases worldwide. Annually, it is estimated that 1.03 million humans become infected, 873,000 develop severe infections, and 49,000 die (Picardeau 2020; Costa et al. 2015). The genome of *Leptospira* is approximately 5000 kb, divided into two 4400 kb and 350 kb sections with GC content of between 35 and 41% (Adler and de la Peña-Moctezuma 2010). The current taxonomic classification, based on whole-genome analysis (pan-*Leptospira*), identified 66 *Leptospira* species that were classified as pathogenic and saprophytic, which were further subdivided into P1 pathogenic, P2 intermediate, and S1 saprophytic (Vincent et al. 2019; Picardeau 2020).

Over 300 *Leptospira* spp. serovars have been identified, which are classified into 28 serogroups based on serological similarity using either the Cross-agglutination-absorption test (CAAT). Serovar classification uses polyclonal antibodies against the bacterial wall lipopolysaccharides (LPS) or the microscopic agglutination test (MAT), a standard test recommended by the World Organization for Animal Health (OIE) (Saito et al. 2013a, b; Bourhy et al. 2013, 2014; Cosate et al. 2017).

The epidemiology of leptospirosis is complex due to the large variety of animal species that can serve as reservoirs and sources of infection for humans and other domestic and wild animals (Andersen-Ranberg et al. 2016). A high percentage of animals become carriers once the bacterium adheres and create replicative niches in the base membrane and luminal sides of the renal proximal tubule epithelial cells, from where it is intermittently excreted into the environment via urine (Yamaguchi et al. 2018).

Colombia has the fourth largest cow herd in Latin America, with a population of 27,973,390 animals, according to the Colombian Agriculture and Livestock Institute (ICA, as its Spanish acronym) (ICA 2021). The high biodiversity and climatic variability of Colombia tropical territory favor silvopasture systems for livestock production but also favor the *Leptospira* presence and proliferation. Leptospirosis, together with other reproductive diseases, creates significant obstacles to increasing the cattle population, sustaining the local demand for meat and dairy products, and improving these goods’ worldwide competitiveness.

This article aims to present state-of-the-art knowledge about the effects of leptospirosis on bovine reproduction and a critical analysis of the research carried out in Colombia. It also aims to promote research and develop guidelines to aid in the development of preventative and control methods within the one-health paradigm.

## Leptospirosis effects on bovine reproduction

Bovine leptospirosis results in economic losses through estrus repetition or the birth of weak calves. It is mainly caused by serovar Hardjo, which has been identified as two species, *L. interrogans* serovar Hardjo (Hardjoprajitno type) and *L. borgpetersenii* serovar Hardjo (Hardjobovis type), with the latter being the most prevalent worldwide (Loureiro and Lilenbaum 2020). The two species are antigenically similar because the *rfb* locus, which contains the genes encoding the proteins involved in LPS biosynthesis, is highly conserved (de la Peña-Moctezuma et al. 2001). Persistent infections caused by serovar Hardjo are related to temporary infertility of variable duration. In contrast, those caused by serovars Pomona and Grippotyphosa manifest as a “storm”—that is, a high number of abortions during a short period of time, accompanied by severe symptoms (Grooms 2006).

The reproductive problems associated with serovars Hardjobovis and Hardjopratjino are difficult to distinguish; serovar Hardjobovis is less adapted to survive in the environment, while serovar Hardjopratjino survives in aquatic environments following excretion via urine (Bulach et al. 2006). Chronically infected animals with serovar Hardjo exhibit low MAT titers (< 1:100) in serum; therefore, these titers (< 1:100) should not be excluded from the farm epidemiological analysis (Sanderson and Gnad 2002).

The bacterium can reach the seminal vesicles and kidneys of bulls infected with serovar Hardjobovis, causing persistent infection and a poor response to treatment, ultimately leading to the animal disposal. *Leptospira* can be vehiculated in semen as it survives at freezing temperatures (Givens 2006; Givens and Marley 2008). PCR test is the most often used method for detecting *Leptospira* contamination in semen (Yang et al. 2018; Júnior et al. 2017). The bacterium pathogenesis in the reproductive tract is unknown, although mediators of the inflammatory response have been identified, which might alter ovulation, early embryonic development, and implantation (Sheldon et al. 2014). Two other theories have been proposed in relation to embryonic loss and estrus repetition: (1) the presence of the bacterium in the uterus causes inflammation, altering the uterine environment and affecting embryo survival or implantation, and (2) direct invasion by the bacterium on the embryo would result in severe damage and subsequent death (Loureiro and Lilenbaum 2020).

A clinical sign of leptospirosis is the so-called milking cow syndrome or milk drop that occurs following a first infection and is mainly associated with the serovar Hardjoprajitno, (Higgins et al. 1980). However, there is no sufficient evidence about the mechanisms that link *Leptospira*, with mastitis and milk production losses. In Argentina and Chile, milking cow syndrome and mortality occurrences in calves have been reported in association to primoinfection with *Leptospira interrogans* serovar Pomona and *L. interrogans* Hardjopratjino (Draghi et al. 2011; Salgado et al. 2015).

The extensive genomic data that are currently available will improve actual molecular typing methods for the entire *Leptospira* genus, including pathogenic and non-pathogenic species worldwide. Genetic variability can be studied in a clinical and environmental context, allowing the identification of new leptospiral mechanisms involved in bacterial virulence and the identification of targets for vaccine development. In Argentina, for example, it was possible to sequence the genomes of *L. interrogans*, *L. borgpetersenii*, *L. biflexa*, and a strain of L. interrogans serovar Pomona, isolated from a bovine abortion outbreak. Such were the first sequences of Argentine isolates (Varni et al. 2016).

Although there is sufficient information on the effects of *Leptospira* at fetal and neonatal levels in cattle, it is scarce in relation to infertility. In a study conducted in Brazil, 33% out of 25 dairy herds with a history of reproductive disorders tested positive against *Leptospira* by group Sejroe, which was strongly associated with estrus repetition (Libonati et al. 2018). The detection of Leptospira hardjo antibodies among ruminants with histories of reproductive disorders in the northeastern part of Nigeria suggests the potential relationships of leptospira with ruminant production system (Stephen et al. 2022).

## Bovine leptospirosis in Colombia

The first studies on *Leptospira* in Colombia were conducted in 1976 in the Colombian highlands by the Animal Health Section of the International Center for Tropical Agriculture, when Anon and collaborators isolated *L. Australis* from kidney tissue of *Proechimys* sp. (spiny rat) and *L. tarassovi* from *Caluromys philander* (opossum) classified by the Pan American Zoonoses Center (CEPANZO) (CIAT 1976). Aycardi et al. (1980) isolated serovar Hardjo from animals that presented low serological titers on a highland farm with abortion events. With this isolation, they conducted infectivity and pathogenicity tests on 5–6-month pregnant seronegative heifers who subsequently delivered weak calves and presented metritis, retained placenta, and leptospiruria lasting between 4 days and 10 months (Aycardi et al. 1982).

The first population-based research in Colombia was conducted using MAT on 1669 bovine sera in three subregions of the Eastern Llanos. Overall seropositivity was 49.1%, with the serovar Hardjo being the most prevalent, followed by Sejroe, Wolffi, and Hebdomadis. The highest prevalence was found in the Llanos Foothills, followed by the Flat High Plains and the Mountain Range (Rivera et al. 1981). Although the authors do not explain these differences, it is suggested that they may be related to the higher humidity in the Llanos Foothills for a large part of the year.

A second population-based research conducted in three subregions in 113 dairy farms using MAT found that seroprevalence was 25.8% overall, 14.4% in the Andean area, 38.2% in the Caribbean, and 24.8% in the Llanos Foothills. The most prevalent serovar was Hardjoprajitno, followed by Pomona, which was significant in the Caribbean region. At a national level, a second population study was conducted 20 years after Griffiths et al. (1982), which used the MAT test and included the same serovars and *L. Borgpetersenii* serovar Hardjobovis (Parra et al. 2002). Table 1 compares the authors’ data for the variation percentage to each serovar in three natural subregions, positive reactor sera, and MAT titers ≥ 1:50. The results of Griffiths et al. (1982) and Parra et al. (2002), as well as the hypothesis, were evaluated using a *Z*-test, and independent proportions were compared in Epidat 4.1 (2012). *L. interrogans* serovar Hardjoprajitno, *L. interrogans* serovar Icterohaemorrhagiae, and *L. interrogans* serovar Grippotyphosa increased, while *L. interrogans* serovar Pomona decreased significantly in the Caribbean Region and the Llanos Foothills.

**Table 1:** Variation in seroprevalence of five *Leptospira* serovars in cows in three natural Colombian subregions, 1980–2000 (MAT titers ≥ 1:50)

	Natural region	Year of the study		Bilateral *Z*-test significance
		1980	2000	
*L. interrogans* serovar Hardjoprajitno	Caribbean	38.9	45.6	0.035
	Llanos Foothills	24.7	47.8	0.000
	Warm Valleys	25.8	38.0	0.000
*L. interrogans* serovar Pomona	Caribbean	15.6	5.0	0.000
	Llanos Foothills	4.6	0.7	0.000
	Warm Valleys	1.2	5.8	0.000
*L. interrogans* serovar Canicola	Caribbean	1.2	2.8	0.071
	Llanos Foothills	4.6	3.4	0.333
	Warm Valleys	1.4	5.6	0.000
*L. interrogans* serovar Icterohaemorrhagiae	Caribbean	0.5	7.5	0.000
	Llanos Foothills	1.3	6.7	0.000
	Warm Valleys	0.3	14.4	0.000
*L. interrogans* serovar Grippotyphosa	Caribbean	0.8	1.2	0.525
	Llanos Foothills	0.0	2.6	0.001
	Warm Valleys	2.8	4.4	0.174

Based on Griffiths et al. (1982) and Parra et al. (2002), the serovar nominated by Griffiths et al. (1982) as Hardjo, corresponded to *L. interrogans* serovar Hardjoprajitno

In 1984, the Colombian–German project (ICA/GTZ) conducted a cross-sectional study in the Department of Córdoba using the MAT test on 104 farms and 2,840 cattle with the serovars Hardjo, Pomona, Canicola, Grippotyphosa, and Icterohaemorrhagiae. The percentage distribution of animals with titers 1:50 and 1:100 by serovar shows that Hardjo, Grippotyphosa, and Icterohaemorrhagiae were the most frequent serovars in animals with titers 1:50 (Otte et al. 1991). In 1993, Corpoica-Ceisa used MAT to analyze 2140 bovine sera and found 681 as positive for serovar Hardjo (32%), 390 for serovar Icterohaemorrhagiae (18.2%), 207 for serovar Pomona (9.6%), and 182 for serovar Canicola (8.5%) (Gallego and Gallego 1994).

A pilot study carried out in 80 farms in the Eastern Llanos that presented abortions and fetal death found a high prevalence of the serovar Hardjo. In two farms, abortion rates reached 36%, with one farm being particularly well-known for abortions occurring in the second trimester of gestation, whereas in the other farm, abortions happened around term, especially during the dry season (Otte et al. 1995). Currently, there is no clear knowledge about the seasonal frequency of abortions in the country.

Seropositivity to *Leptospira* spp. was 92% in breeding bulls from 11 farms with other reproductive diseases in the Sabana, Bogotá, and was distributed as follows, Pomona (62%), Canicola (62%), Hardjopratjino (38%), Grippotyphosa (23%), and Icterohaemorrhagiae (18%); copositivity to more than one serovar was Canicola, Pomona (48%), Hardjo, Pomona (23%), Hardjo, Canicola (15%), and Hardjo, Canicola, and Pomona (15%) (Góngora et al. 1995). It is important to recognize how little we know about the epidemiological importance of these serovars and how different cross-reactions between serovars can affect an individual or a herd. The same situation occurs with copositivity with other bacterial, viral, and parasitic infectious agents. It is possible that, under the current epidemiological situation of the country, *Leptospira* infection is highly associated with other infectious agents.

A serological sample was taken from 205 *Bos indicus* cows from the breeding production systems of the High Plains and the Llanos Foothills and 175 of their fetuses at the Villavicencio slaughterhouse. The cows showed seroreactivity to the MAT test, ≥ 1:25, 53.6%, 52.7%, 34.9%, 29.6%, and 6.4% to the serovars Canicola, Hardjoprajitno, Pomona, Icterohaemorrhagiae, and Grippotyphosa, respectively, and seroreactivity for the same serovars in fetuses with titers ≥ 1:10 were 11.3%, 17.36%, 16.0%, 6.5%, and 5.9%. The median serological titer in cows was 1:400 for Hardjoprajitno, 1:100 for Grippotyphosa, and 1:50 for Canicola, Pomona, and Icterohaemorrhagiae. In contrast, the median titer in fetuses was 1:80 for Hardjoprajitno, 1:40 for Grippotyphosa, and 1:10 for Canicola, Pomona, and Icterohaemorrhagiae (Barrera and Castaño 1998). The same study detected Hardjoprajitno antigen by indirect immunoperoxidase (IPI) in 12% of the fetuses’ kidneys and 5.2% of their livers, suggesting transplacental and thus fetal infection by *Leptospira*. It is clear that immunoglobulins cannot pass through the bovine epitheliochorial placentation, and the presence of *Leptospira* indicates an immune response to fetal infection, evidenced by the presence of *Leptospira* antigen in the tissues.

In Antioquia, 722 sera were analyzed, finding a prevalence of 21.7%; the highest seropositivity corresponded to serovar Bratislava (48.5%), followed by serovar Hardjo (30.5%). Sera from 214 breeding pigs were analyzed in the same farms, finding seropositivity of 25.7% with serovars Bratislava, Pomona, and Canicola. It was shown that the higher prevalence of serovar Bratislava in cattle was associated with the use of pig manure to fertilize the paddocks, which favors the infectious cycle of *Leptospira* between the two species (Ochoa et al. 2000). A recent study in Brazil in a bovine and swine mixed system found a high calving interval in cattle (> 450 days). Although *Leptospira* was diagnosed in both species, no direct association was found between the two production units; however, it is suggested that the differential diagnosis of *Leptospira* should be established when reproductive disorders and spontaneous abortion are present using lower cut-off points to establish a better diagnosis at herd level (Mori et al. 2017).

Corpoica carried out a new seroprevalence study in 2000, using 6 serovars of *Leptospira* sp., in 5 natural subregions, 15 micro-regions, two transects, 6590 cattle, in 249 farms, and the dual-purpose bovine production system of the Colombian low tropic. A positive reactor serum was defined as one that agglutinated 50% or more of a *Leptospira* sp. culture at a dilution of ≥ 1:50 (Parra et al. 2002). Two thousand nine hundred forty-nine (44.7%) of the 6590 bovine sera evaluated with MAT showed positive seroreactivity to one or more *Leptospira* serovars. Hardjoprajitno was the most prevalent serovar in the five subregions, with an overall positive reactivity of 36.3%. The Inter-Andean valleys’ subregion had a significantly lower proportion of positive reactors than the other subregions, which all had similar proportions (Table 2).

**Table 2:** Seroprevalence of 6 *Leptospira* serovars in 5 regions and 17 subregions in Colombia. Dual-purpose bovine systems

Regions and subregions	(*n*)	*Leptospira* serovars (seroprevalence %)					
		Hardjoprajitno	Hardobovis	Pomona	Canicola	Icterohaemorrhagiae	Grippotyphosa
***Total*** ***CI: 95%***	***6590***	***36.3*** *35.2–37.5*	***5.2*** *4.7–5.7*	*4.3* *3.9–4.9*	*3.9* *3.5–4.4*	*9.0* *8.3–9.7*	3.1 *2.7–3.6*
**Western Caribbean**	**1392**	**41.2**^**a**^	**2.9**^**d**^	**5.4**^**b**^	**3.4**^**c**^	**10.4**^**a**^	**1.5**^**c**^
Momposina Depression	154	53.2^a^	1.3^a^	3.9^a^	1.9^a^	3.2^b^	1.9^ab^
Sinú Valley	120	48.3^ab^	2.5^a^	3.3^a^	2.5^a^	7.5^ab^	0.0^b^
Savannas of the Caribbean	628	39.3^ab^	3.5^a^	5.6^a^	4.6^a^	12.3^a^	0.6^b^
Coastal Strip	409	38.9^ab^	2.7^a^	6.4^a^	3.2^a^	12.2^a^	3.2^a^
Lower Cauca	81	33.3^b^	1.3^a^	4.9^a^	0.0^a^	4.9^b^	1.2^ab^
CI: 95%		38.6–43.8	2.2–4.0	4.3–6.7	2.0–3.7	8.9–12.1	1.0–2.3
**Eastern Caribbean**	**1165**	**39.7**^**a**^	**1.0**^**e**^	**4.5**^**b**^	**2.1**^**c**^	**5.2**^**b**^	**0.9**^**c**^
Lower Magdalena	461	43.8^a^	0.7^a^	4.8^a^	2.4^a^	4.3^a^	0.4^a^
Cesar Valley	360	36.9^a^	1.4^a^	6.9^a^	1.4^a^	5.0^a^	1.1^a^
Central and Southern Cesar	344	36.9^a^	1.2^a^	1.5^b^	2.3^a^	6.7^a^	1.2^a^
CI: 95%		36.9–42.5	0.6–1.8	3.4–5.8	2.0–3.7	4.1–6.7	0.5–1.6
**Llanos Foothills**	**1502**	**37.5**^**a**^	**5.2**^**c**^	**0.9**^**c**^	**2.7c**	**4.9**^**b**^	**2.6**^**b**^
Arauca Foothills	494	43.3^a^	5.1^b^	1.0^a^	2.2^b^	4.3^b^	5.3^a^
Casanare Foothills	334	31.4^b^	10.5^a^	0.3^a^	5.4^a^	8.4^a^	0.3^c^
Meta Foothills	674	36.3^b^	2.7^c^	1.2^a^	1.8^b^	3.7^b^	1.8^b^
CI: 95%		35.1–40.0	4.2–6.4	0.6–1.6	2.0–3.7	3.9–6.1	8.5–13.3
**Amazon Foothills**	**641**	**37.1**^**a**^	**11.0**^**a**^	**9.7**^**a**^	**7.6**^**a**^	**12.3**^**a**^	**10.6**^**a**^
Florencia-San Vicente	522	39.3^a^	12.1^a^	11.9^a^	8.7^a^	14.1^a^	12.2^a^
Morelia-Albania	119	27.7^b^	6.0^b^	0.0^b^	2.6^b^	4.3^b^	3.4^b^
CI: 95%		33.5–40.9	8.7–13.6	7.6–12.3	5.8–9.9	10.0–15.1	8.5–13.3
**Inter-Andean Valleys**	**1890**	**29.4**^**b**^	**7.4**^**b**^	**4.4**^**b**^	**5.1**^**b**^	**12.4**^**a**^	**3.7**^**b**^
High Magdalena Valley	488	21.9^b^	5.3^ab^	5.4^a^	2.7^b^	14.1^ab^	1.6^b^
Middle Magdalena Valley	618	29.8^ab^	14.7^a^	3.1^ab^	9.9^a^	12.1^ab^	8.1^a^
Cauca River Valley	580	34.8^a^	2.8^b^	6.0^a^	1.9^b^	8.1^b^	1.4^b^
Patía Valley	204	30.9^ab^	3.4^b^	1.5^b^	5.4^a^	21.1^a^	1.5^b^
CI: 95%		27.4–31.5	6.3–8.7	3.6–5.4	4.2–6.2	4.2–6.7	2.9–4.6

In the columns, seroprevalences between regions (in bold) and subregions of the same region that do not share the superscript letter for the same serovar are statistically different. Significance *P* < 0.05. *CI*, confidence interval of the total proportion by serovar. χ^2^ Chi-square test of independence between subregions. The values ​​in bold correspond to the total value of *n* by region and average values ​​of the seroprevalence of the different serovars by region

Icterohaemorrhagiae was the second most prevalent serovar, followed by Hardjobovis. The degree of infection was significantly different in each subregion. For each Hardjobovis seropositive cattle, 7 were found seropositive for Hardjoprajitno; Grippotyphosa was the least prevalent of the 6 serovars.

The proportions of positive seroreactors by age group were statistically equal in calves, heifers, cows, and bulls. For serovars Hardjoprajitno, Hardjobovis, and Canicola, the age groups were independent of the variation of positive seroreactors. There were substantial differences in the proportion of seropositive animals between serovars Icterohaemorrhagiae and Grippotyphosa, with the proportion of seropositive calves being much greater (Table 3).

**Table 3:** General seroprevalence of six *Leptospira* serovars by age group. Dual-purpose bovine systems

Age group	*Leptospira* serovars (seroprevalence %)					
	Hardjoprajitno	Hardjobovis	Pomona	Canicola	Icterohaemorrhagiae	Grippotyphosa
Calves	36.2^***a***^	4.7^***a***^	4.5^***ab***^	4.6^***a***^	10.0^***a***^	3.0^***ab***^
Heifers	38.0^***a***^	4.5^***a***^	5.5^***a***^	3.3^***a***^	9.7^***ab***^	1.9^***b***^
Cows	36.3^***a***^	5.7^***a***^	4.0^***ab***^	3.8^***a***^	8.3^***ab***^	3.8^***a***^
Bulls	31.1^***a***^	5.1^***a***^	2.6^***b***^	2.6^***a***^	6.4^***b***^	1.9^***b***^
*P* value	0.158	0.295	0.054	0.157	0.057	0.006

Source: Parra et al. (2002). *N*, number of determinations. Columns with different letters present different statistical proportions. *CI*, confidence interval of the total proportion by serovar. χ^2^ Chi-square test of independence

**Fig. 1:**
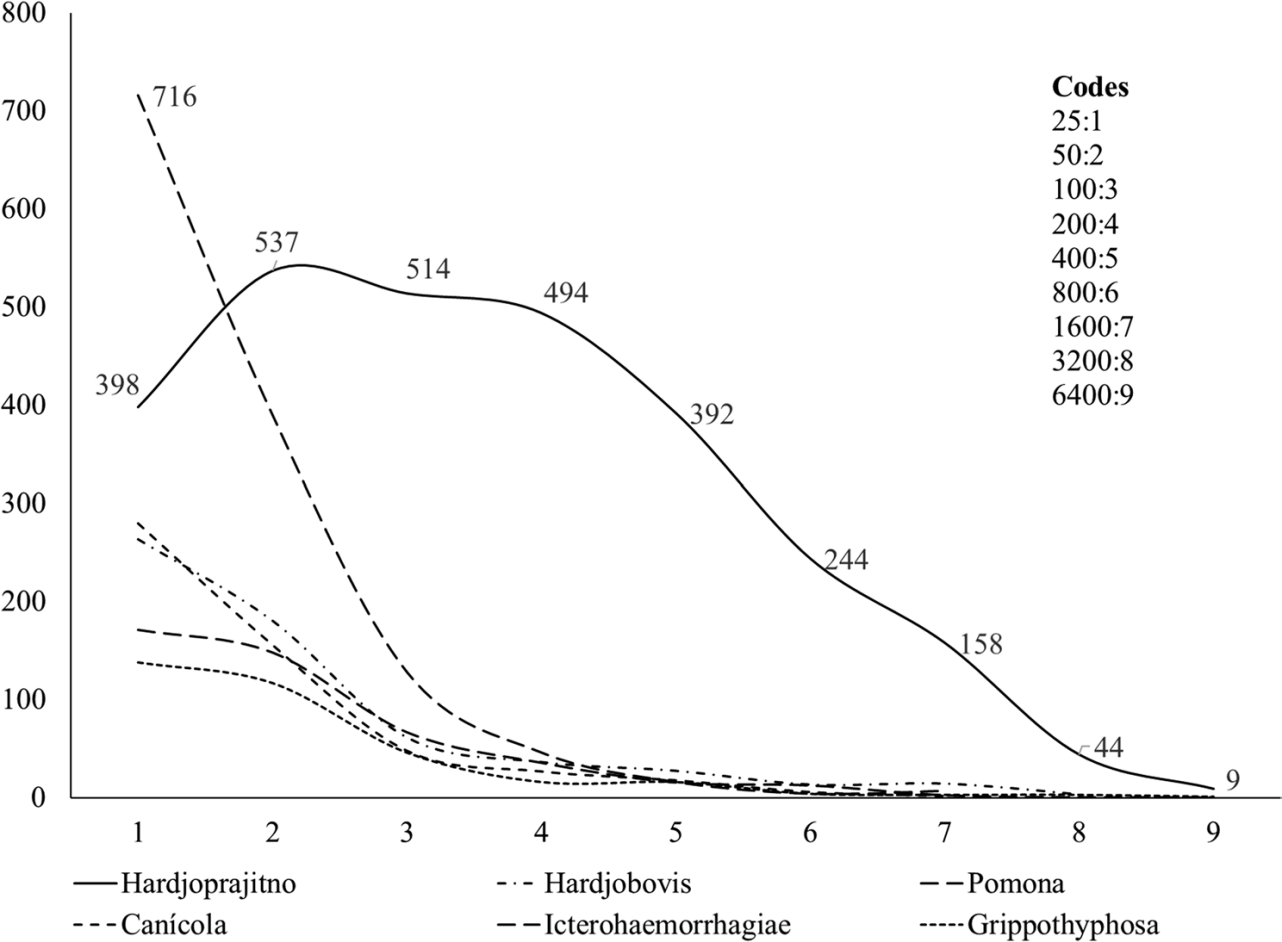
Percentage distribution of titers to six *Leptospira* serovars in cattle of the dual-purpose system in Colombia. Colombian Tropical Lowlands. Source: Parra et al. (2002)

An adequate spectrum is established by considering the number of sera tested; the diversity of cattle origins, subregions, micro-regions, farms, and age groups; and the titration of sera at 1:25 dilution. Although the zero titer corresponds to a 1:25 negative, it was not included in the titer display options. It is also the most frequent titer across all serovars by regions, subregions, and age groups. Based on the above, Parra et al. (2002) developed a frequency polygon of the titers to six *Leptospira* serovars (Fig. 1).

All serovars except Hardjoprajitno had the highest titer of 1:25. Titers 1:50 are relatively visually identical for serovars Hardjobovis, Pomona, Canicola, and Grippotyphosa. Titers of 1:100 and 1:200 are scarce for these serovars, including Icterohaemorrhagiae, which is helpful since the dilution of the serum 1:50 is regarded as adequate interaction to establish innate, acquired immunity. Cattle are considered the maintenance of Hardjoprajitno, which demonstrated a titer distribution that is significantly different from the other serovars (*P* < 0.001 goodness of fit), with a plateau at 1:50, 1:100, and 1:200, following a lower frequency with a titer of 1:25, and then a progressive decrease, where each dilution reduces the frequency by an average of 100 observations.

In the Colombian coffee region, between 2002 and 2005, MAT was used to evaluate 1789 cattle from 158 farms. An accumulated seroprevalence of 16.4% in animals and 32.5% in farms was found. The predominant serovar was Hardjo with 45.7%, followed by Grippotyphosa with 18.9%, Canicola with 14.5%, Icterohaemorrhagiae with 9.1%, Pomona 6.8%, and Bratislava with 5.0%. The risk factors found for seroreactivity were the presence of rodents, proximity to garbage dumps, humid climate zones, presence of wetlands in paddocks, inadequate disinfection of the premises, and the presence of horses on the farms (Zuluaga 2009).

In the Department of Caquetá, a prevalence of antibodies to leptospirosis was found in buffaloes 37.3% and bovines 28.0%, detecting the presence of antibodies to serovars Bratislava, Canicola, Grippotyphosa, Hardjo, Pomona, and Icterohaemorrhagiae, with serovars Grippotyphosa and Hardjo being the most prevalent. Although seropositivities for *Leptospira* were higher in single-species herds, serovar Hardjo was significantly higher in sampling sites where both species were present (Motta et al. 2014). In some Colombian regions (Caquetá, Meta, Antioquia), mixed farms are typical, but they have received little attention from the infectious point of view in relation to *Leptospira* and other infectious agents.

In a study of the serological dynamics in dual-purpose cows from the municipality of Guamal, Meta, the serovar with the highest prevalence was Tarassovi (90.2%), followed by Hardjopratjino (80.5%) (Ordoñez et al. 2017). The authors recollected 440 samples from 23 municipalities of the Department of Santander using the double antibody ELISA, which detected Hardjobovis and Hardjopratjino with a prevalence of 26.1% (Vargas-Niño et al. 2018). In general, it has been shown in other countries that *Leptospira* serovar diversity and prevalence vary widely depending on latitude, region, environmental conditions, the presence of wild reservoirs, and the time of year in which the study was conducted; new studies are therefore needed to more precisely define the leptospirosis situation at the national level.

There has been little research on leptospirosis in the country from a public health standpoint. Seroprevalence is between 15 and 20.7% in the seemingly healthy human population, and among occupational risk groups, seroprevalence ranges from 7% in slaughterhouse employees to 35% and 48% in pig and fish farm workers, respectively (Góngora et al. 2008). According to the Colombian National Institute of Health (INS), in Colombia during 2018, 2137 cases of leptospirosis were reported. According to the type of case, these were classified as 1570 (73.5%) as suspects, 528 (24.7%) confirmed by laboratory, and 39 (1.8%) confirmed by epidemiological link. Probable deaths from leptospirosis were 52, of which 6 were confirmed (INS 2020).

## Future perspectives

Bovine leptospirosis is one of the reproductive diseases that causes the most economic losses in the cattle industry; however, these losses have not been fully quantified in the country. Seroprevalence studies indicate that seropositivity has increased significantly over time in the various cattle-raising regions tested, implying an endemic presentation. However, it is unknown when most sporadic cases occur. It is necessary to initiate isolation and molecular characterization studies of the various serovars by region, which will allow for advancements in vaccine production using national field strains that may provide improved immunity. Simultaneously, clinical characterization of the “milk drop syndrome” would aid determining its actual involvement in bovine abortion and distinction from other causes of abortion.

Given its zoonotic origin, it is critical to focus on issues like diagnosis, as the disease may be underdiagnosed in humans due to the complications involved. In addition, due to the country’s high sensitivity to environmental consequences, especially climate change, leptospirosis is one of the illnesses to be considered.

Advances in molecular biology techniques, combined with the current availability of whole genomes for several *Leptospira* strains in the GenBank database, will enable the study and characterization of isolations at the national level using techniques such as repeated sequence analysis (MLVA) and phylogenetic analysis based on specific gene sequences. With regard to those mentioned above, there is a research gap in comparison to countries in the southern cone, such as Argentina, Brazil, Chile, and Uruguay (Cosate et al. 2017; Zarantonelli et al. 2018; Koval et al. 2020; Luna et al. 2020), where significant progress has been made in the molecular characterization of circulating *Leptospira* strains.

## Conclusions

It is evident that the government must enhance productivity indices in livestock production and expand the existing cattle population, both of which are complicated by leptospirosis**.** In addition, there is limited information about the genetic, molecular, and epidemiological aspects of circulating serovars. This study highlights the need to (1) conduct isolation studies, molecular characterization, and genomic sequencing of serovars circulating in Colombia, as well as the differences in clinical outcomes among serovars to better understand the health impact in cattle farming, and (2) to assess the economic impact of leptospirosis in livestock production.

## Data Availability

No datasets were generated or analyzed during the current study.

## Code availability

No applicable.
